# Social network and extension service in farmers’ agricultural technology adoption efficiency

**DOI:** 10.1371/journal.pone.0235927

**Published:** 2020-07-10

**Authors:** Geling Wang, Qian Lu, Sergio C. Capareda

**Affiliations:** 1 College of Agricultural and Life Science, Texas A & M University, College Station, TX, United States of America; 2 College of Economics, Xi’an University of Finance and Economics, Xi’an, China; 3 College of Economics and Management, Northwest A & F University, Yangling, China; Institute for Advanced Sustainability Studies, GERMANY

## Abstract

The purpose of the article is to analyze the interaction effect between social network and extension service in farmers’ agricultural technology adoption efficiency (TAE). The empirical analysis refers to samples of farmers’ water-saving irrigation technology (WSIT) adoption from Minqin, China. The result indicates that social network and extension service can improve farmers’ TAE, but they are found to be competitive from the perspective of overall social network. While from the perspective of four dimensions of social network, interaction and trust have positive effects on farmers’ TAE, and learning and trust are found to be competitive with extension service. The policy implication describes as follows. First, government should pay attention to farmers’ social learning and demonstration effect. And series of measures should be taken to cultivate farmers’ social network. Second, extension service should be improved to satisfy farmers’ multiple demand on agricultural technology. Third, government should combine the effects of social network and extension service, and make different promotion strategies for different regions. In addition, other influential factors cannot be ignored.

## Introduction

The promotion of new agricultural technologies is the basis way to agriculture transformation. While in many developing countries, the effect of government’s extension service is not that much ideal. New agricultural technologies can’t be understood and accepted by most farmers, which results in low efficiency of agricultural technology and leads to the low speed of agriculture development. In fact, a key factor during farmers’ agricultural technologies adoption has always been ignored. That is social network. According to recent studies, famers’ technology adoption can easily be affected by the behaviors of others among their social network [1, 2].

Since both social network and extension service are important in farmers’ technology adoption efficiency, what is the relationship between them? Does social network (informal organization) function as substitution or complementation to extension service (formal organizations)? The answers to these questions are very important to understand the influential mechanism of social network in the process of farmers' adoption.

Therefore, the purpose in this paper is to figure out the interactive relationship between social network and extension service in farmers’ technology adoption efficiency (TAE). The rest of the article is organized as following: section 2 is a literature review. Section 3 describes the survey and the data. Section 4 establishes a model to analyze the relationship between social network and extension service. In section 5, we discuss the results. And the last part presents conclusion and discussion.

## Literature review

Social network and extension service are two main approaches to the diffusion of agricultural technology, and they are also the two main channels for farmers to acquire technical information. Social network emphasizes farmers using their social relations to transmit technical information and interacted with each other. Farmers’ technology adoption is a dynamic learning process [2]. Farmers obtain technical information through social interaction, fix the expected return of technology and make adoption decision. Social network has the function of providing information, reducing risks and making up for defects in the formal system [3]. In an area that is characterized by obvious social network with the kinship, the geographical relationship and the industry-predestined relationship, social network with the principle of closeness plays an important role in farmers’ technology adoption.

While extension service emphasizes the government's intervention, control and institutional links, and plays a major role in the promotion of agricultural technology. However, for a long time, it is difficult for extension agencies to adapt to the diversified technology needs of farmers under the market economy, which leads to the short supply of agricultural technology and the low efficiency of extension service.

There may be a complex relationship between extension service and social network [2,4]. Goyal and Netessine believes that by establishing a "model household", relying on a core member to disseminate information can reduce technology uncertainty and improve the efficiency of technology utilization [5]. However, it is not yet clear whether there is any relationship between extension service and social network in technology adoption. Duflo et al. find that there is no sufficient evidence of social learning when promoting technology by extension service [4]. While through the empirical research using the agricultural irrigation technology, Genius et al. find that extension service and social network are strong determinant factors in a technology’s promotion and adoption, and the effectiveness of the two information channels is enhanced by the presence of the other party [2].

Previous studies of agricultural technology promotion and adoption paid little attention to the interaction effect of social network and extension service. The problems of low technology efficiency and weak response to farmers' demand [6] have not been effectively solved for a long time. In addition, social network has rich connotation, while the existing researches often explain with one dimension to study its effect [7–9], which result to different or even contradictory conclusions.

Therefore, taking the example of farmers’ water-saving irrigation technology (WSIT) adoption data in Minqin, China, we present evidence on how social network and extension service interact to farmers’ technology adoption effect. We hope the research can provide new ideas for developing countries to promote agricultural technology

## The survey and the data

### The survey

We study farmers’ WSIT adoption in Minqin region of China. Minqin is located in the north of Hexi Corridor and the lower reaches of Shiyanghe River. Three sides of Minqin were surrounded by Badain Jaran Desert and Tengger Desert. It is famous for temperate continental arid climate and continental desert climate. The typical climate characteristics make the local farmers have strong awareness of the scarcity of water resources. So it is urgent to find ways to improve water resources utilization efficiency.

The practice has proved that WSIT has the function of improving water resources utilization efficiency, reducing drought losses, reducing rural poverty and promoting agricultural change [9]. It is of great practical significance to promote WSIT in Minqin. In fact, the Chinese government has taken a series of measures to promote farmers to adopt WSIT in this area. China began to implement a pilot program for water-saving society construction in Minqin as early as 2004. WSITs, such as pipe conveyance drip irrigation, tubular outflow, ridge irrigation, ridge furrow irrigation, ridge film furrow irrigation, plastic film recycling etc. were actively promoted to improve the efficiency of water resources utilization effectively, through training, demonstration and field guidance etc. As of 2018, the efficient water-saving irrigation area in Minqin amounted to 39.88 thousand acres.

Therefore, we took stratified sampling method and conducted a household survey in Minqin from October to November in 2018. Our survey is anonymous, and the data is used for scientific research only. The information and privacy of the respondents are protected. A total of 300 questionnaires were sent out and returned. After taking out the incomplete or obviously inconsistent questionnaires, we obtained 278 valid samples (Table 1).

**Table 1:** Village description.

	A	B	C	D	E	F	G	H	I	J
Sample number	27	34	26	24	36	25	21	33	23	29
Approximate household number ^a^	250	300	250	250	350	250	200	300	200	250
Distance from Hongyashan Reservoir (km)^b^	35	30	30	40	30	40	50	35	40	50
Distance from nearest market(km)	3	3	4	4	1	3	3	3	3	2
Distance to the town (km)	7	3	4	7	2	8	11	3	8	10
Extension service ^c^	**√**	**√**	**√**	**√**	**√**	**√**	**√**	**√**	**√**	**√**
Water users association ^d^	√	√	√		√	√		√	√	√

^a^ Household number of a village is a approximate figure.

^b^ The distance is the way from village committee to the destination.

^c^ Extension service means that the government has promoted WSIT in the village.

^d^ At the survey time, water users associations were operating in many villages.

### The data

#### Social network

In the existing literature, individual variables are selected as evaluation index of social network sometimes, such as the number of contactors in holidays [10], and the spending on interpersonal interaction per year [11], and sometimes multiple indicators are selected, for example, Liu divided social network into three dimensions, including networks size, networks resources, and interaction frequency [12]. The normal practice of calculating social network is processing the original indexes with Factor Analysis Method. The bigger the value of social network, the higher farmer’s social network is. In this study, we defined that social network is a kind of resources which based on ones’ social interpersonal communication, safeguarded by the rules and regulations formed by the long-term group activities, operated by group members’ learning, interaction, reciprocity and trust. According to a recent literature study by Wang and Lu [13], we adopt nine variables which represent four aspects of social network as evaluation system of social network (Table 2).

**Table 2:** Variables of social network.

Variable		Description	Mean	SD
**Learning**				
	Frequency of communicating with others about technology using	never = 1->5 = frequently	2.84	1.03
	Frequency of consulting model household about technology matters	never = 1->5 = frequently	2.56	1.21
	Frequency of visiting demonstration farmland	never = 1->5 = frequently	2.3	1.29
**Interaction**				
	Frequency of activities with regular technology adopters	never = 1->5 = frequently	1.94	0.89
	Frequency of activities with adopters who are intimate friends with each other	never = 1->5 = frequently	2.23	0.90
**Reciprocity**				
	Everyone is willing to help when some general events happens	strongly disagree = 1->5 = strongly agree	4.1	0.59
	There are a lot of people who help out during difficult time	strongly disagree = 1->5 = strongly agree	3.84	0.71
**Trust**				
	I'm willing to lend something to people around me	strongly disagree = 1->5 = strongly agree	4.21	0.60
	Neighborhood relations are very harmonious	strongly disagree = 1->5 = strongly agree	3.53	0.82

#### Learning

Learning exists in individuals’ daily life whenever and wherever possible, and they make progress through learning. Specifically, there are three kinds of learning during farmers’ technology adoption: consulting model household about technology matters, imitating how other farmers doing, and communicating and sharing with each others.

#### Interaction

According to the extent of interaction, it can be divided into two kinds: general interaction and deep interaction. General interaction refers to the correspondence among regular adopters, while deep interaction involves activities among adopters who are intimate friends with each other.

#### Reciprocity

There are two kinds of reciprocity: reciprocity in general moment and reciprocity at crucial moment. The former refers to the help occurs when some general events happens, such as weddings or funerals et al., while the latter represents the help when dealing with some insurmountable difficulties.

#### Trust

Individual trust in strangers refers to the degree whether individuals are willing to lend something to others, and mutual trusts can be measured by the evaluation of the perception of individual trust with each other.

#### Extension service

Two sides can measure extension service: the ways of extension service and the effect of extension service (Table 3). Specifically, the ways of extension service are mainly refer to collective technical training and field guidance, which are the two main ways of extension service. And the effects of extension service involve three aspects: the difficulty level, the mastery level, and the helpfulness rate.

**Table 3:** Variables of extension service.

Variable		Description	Mean	SD
**Ways of extension service**				
	Frequency of participation in collective technical training	never = 1->5 = frequently	2.78	0.41
	Frequency of participation in field technical guidance	never = 1->5 = frequently	2.66	0.47
**Effects of extension service**				
	It's easy for me to understand the content of government's extension service	strongly disagree = 1->5 = strongly agree	3.84	0.62
	It's easy for me to master the skill of WSIT through extension service	strongly disagree = 1->5 = strongly agree	3.71	0.71
	Government's extension service is of great help to agricultural production	strongly disagree = 1->5 = strongly agree	3.05	0.92

#### Other variables

Previous studies have shown that factors such as sex, age, education level, length of farming, water price, cultivated land quality etc. affect farmers’ efficiency of WSIT adoption. In addition, factors such as water users association [14], water resource scarcity awareness [15], water environment [16], technology cognition [17] cannot be ignored. Therefore, we select the following indicators as the factors affecting farmers’ WSIT adoption efficiency (Table 4).

**Table 4:** Influential factors of farmers’ WSIT adoption efficiency.

Variable	Description	Mean	SD
extension service (ES) ^a^	[0,1]	0.58	0.19
social network (SN)	[0,1]	0.43	0.18
**four dimensions of social network**			
learning	[0,1]	0.36	0.20
interaction	[0,1]	0.39	0.18
reciprocity	[0,1]	0.66	0.15
trust	[0,1]	0.56	0.17
**other factors**			
sex	sex: male = 1, female = 0	0.72	0.45
age	age (years)	52.44	8.88
education	education level: illiterate = 1,primary school = 2,middle school = 3,high school = 4,college and above = 5	3.33	1.05
water users association	Do you join the water users association?	0.37	0.48
	yes = 1,no = 0		
water resource scarcity awareness	Wells are getting deeper and deeper.	3.78	1.15
	strongly disagree = 1->5 = strongly agree		
water-using environment	Water theft is becoming less and less common in the village.	3.41	0.93
	strongly disagree = 1->5 = strongly agree		
water price	Water price is getting higher and higher.	2.01	1.34
	strongly disagree = 1->5 = strongly agree		
technology cognition	WSIT is becoming more and more important to ensure agricultural production.	3.24	0.92
	strongly disagree = 1->5 = strongly agree		

^a^ Factor Analysis Model was used to measure social network and extension service.

## Theoretical model

### Calculating technology adoption efficiency

To analyze the interactive effects of social network and extension service on farmers’ technology adoption efficiency (TAE), we should first calculate farmers’ TAE. As we know that there are two major approaches of measuring efficiency. They are SFA (Stochastic Frontier Analysis) and DEA (Data Envelopment Analysis). The biggest difference is that DEA is a non-parametric method, while SFA is a parametric method. In our study, we need to establish a two steps measurement to calculate famers’ TAE. So SFA was chosen as the first step to calculate farmers’ production technical efficiency so that we can use its stochastic frontier production function to establish a Single Factor Input Model to calculate TAE. This method was used in many papers, such as Wang and Li [14], Wang and Zhao [18], Xu and Huang [17] etc.

#### Measuring farmer’s production technical efficiency

Let assume the stochastic frontier production function has the following form:

$$Y_{i}=x_{i}\beta +\left(V_{i}-U_{i}\right)\;i=1,2,\cdots n$$ (1)

Where *Y*_*i*_ and *x*_*i*_ are the output and input vector of farmer *i*, *β* is the corresponding parameters vector, *V*_*i*_ is the random error and obeys normal distribution which mean value is zero and variance is $\sigma_{V}^{2}$. *U*_*i*_ is a nonnegative random variable and represents the technique inefficiency in production, it follows the truncated normal distribution which standard deviation is *σ*_*U*_. *V*_*i*_ is independent of *U*_*i*_. So farmers’ production technical efficiency can be written as [19]:

$$TE_{i}=\exp\left(-u_{i}\right),$$ (2)

Assuming that $\sigma^{2}=\sigma_{V}^{2}+\sigma_{U}^{2},\;\gamma =\sigma_{U}^{2}/\sigma^{2}$, then the value of *γ* is between 0 and 1. Technical loss mainly comes from random error term when the value of *γ* is closer to 0, and it mainly comes from technical invalidity when *γ* is closer to 1.

#### Measuring farmer’s TAE

TAE is calculated by using Single Input Efficiency Model. The efficiency of a single input factor is the ratio between the optimal input and the actual input when the output and other inputs are given. Take WSIT as an example, the efficiency of a single input factor (water) can be written as:

$$WE=\left[\min\left\{\lambda :f\left(x,\lambda_{w};\alpha \right)\geq Y\right\}\right]\to (0,1]$$ (3)

Where *WE* is farmers’ TAE, *w* is the actual water consumption, *x* are the other inputs except water, *λ* is the ratio between the optimal water usage under the condition of fully technical efficiency and the actual water consumption, *λ*_*w*_ is the optimal water usage, and *α* is the input coefficient. When measuring TAE, the first thing to be consider is estimating *λ*_*w*_. Using the efficiency loss model of Battese and Coelli [20],the model of production function without efficiency loss can be write as:

$$Y_{i}=kw^{*}+x_{i}\beta +V_{i}$$ (4)

Where w* is the optimal water usage. By solving simultaneous equations $\{\begin{array}{l} Y_{i}=kw^{*}+x_{i}\beta +V_{i}\\ Y_{i}=kw_{i}+x_{i}\beta +\left(V_{i}-U_{i}\right) \end{array}$ and estimating the parameters, TAE is calculated [21].

#### Estimating influencing factors of TAE

Farmers’ TAE can be calculated by the above two steps, and then the efficiency influential factor model can be written as:

$$WE_{i}=z_{i}\delta +e_{i}$$ (5)

Where *WE*_*i*_ and *z*_*i*_ are TAE of farmer *i* and the influential factors vector, *δ* is the corresponding parameters vector, *e*_*i*_ is an independent identically distributed random variable with a mean value of 0 [22].

### Effect of technology adoption efficiency

According to the theoretical analysis and the influential factors selection, we can write the influential factors analysis model as:

$$\{\begin{array}{l} WE=\delta +aES+bSN+\sum_{j}\gamma_{j}z_{j}+e\;j=1\cdots 8\\ WE=\delta +aES+\sum_{i}b_{i}SN_{i}+\sum_{j}\gamma_{j}z_{j}+e\;i=1\cdots 4\;j=1\cdots 8\\ WE=\delta +aES+bSN+cES*SN+\sum_{j}\gamma_{j}z_{j}+e\;j=1\cdots 8\\ WE=\delta +aES+\sum_{i}b_{i}SN_{i}+\sum_{i}c_{i}ES*SN_{i}+\sum_{j}\gamma_{j}z_{j}+e\;i=1\cdots 4\;j=1\cdots 8 \end{array}$$ (6)

Where *WE* is farmer’s TAE, *ES* and *SN* are social network index and extension service index, *SN*_*i*_ are four dimensions indexes of social network, and *z*_*j*_ are other influential factors, *δ*,*a*,*b*,*c*,*b*_*i*_,*c*_*i*_,*γ*_*i*_ are parameters to be estimated.

Farmers’ TAE is bounded variable and its value is between 0 and 1, the variable does not appear to be normal distribution. If the ordinary least square method (OLS) is adopted directly, the result will be biased. So we choose Tobit model for the analysis.

We standardized social network index, extension service index and the four dimensions of social network. After that, the values of the index are between 0 and 1. So the variables can be compared in magnitude, and it is beneficial to compare and analyze the effect of social network and extension service on farmer’s TAE.

When analyzing the interaction relationship between social network and extension service, we added the cross items. In order to prevent multicollinearity between the cross terms and the original variables, we centralized the original data.

## Empirical results

### Farmers’ technology adoption efficiency

According to the theoretical models, we firstly calculated farmers’ production technical efficiency. Using beyond logarithmic stochastic frontier production function, the model of production function can be written as:

$$\begin{array}{l} \ln y_{t}=\beta_{0}+\beta_{1}\ln w_{t}+\beta_{2}\ln L_{t}+\beta_{3}\ln C_{t}\\ \;+\beta_{4}{\left(\ln w_{t}\right)}^{2}+\beta_{5}{\left(\ln L_{t}\right)}^{2}+\beta_{6}{\left(\ln C_{t}\right)}^{2}\\ \;+\beta_{7}{\left(\ln w_{i}\right)}^{*}\left(\ln L_{i}\right)+\beta_{8}{\left(\ln w_{i}\right)}^{*}\left(\ln C_{i}\right)+\beta_{9}{\left(\ln L_{i}\right)}^{*}\left(\ln C_{i}\right)+v_{i}+u_{i} \end{array}$$ (7)

Where in the equation, *y*_*i*_, *w*_*i*_, *L*_*i*_ and *C*_*i*_ are the output earnings, irrigation water cost, labor input and capital input per unit area of farmer *i* respectively. And the capital input is the sum of the seed, fertilizer, pesticide, machinery, and plastic film in a unit area. *β* is the parameter to be estimated. Then farmers’ TAE can be calculated by the following formula:

$$WE_{i}=\exp\left(\left(-\varsigma_{i}\pm \sqrt{\varsigma_{i}^{2}-2\beta_{4}u_{i}}\right)/\beta_{4}\right)$$ (8)

In the formula, *ς*_*i*_ can be calculated by the following formula: $\varsigma_{i}=\frac{\partial \ln y_{i}}{\partial \ln w_{i}}=\beta_{1}+2\beta_{4}\ln w_{i}+\beta_{7}\ln L_{i}+\beta_{8}\ln C_{i}$.

We estimate the logarithmic stochastic frontier production function and the results are shown in Table 5.

**Table 5:** Results of stochastic frontier production model.

Variable		Coef.	Std.Err.	Variable		Coef.	Std.Err.
_cons	β_0_	2.4085* ^a^	0.9987	L^2^	β_5_	-0.0247	0.0443
w	β_1_	0.3436*	0.1857	C^2^	β_6_	-0.0270	0.0255
L	β_2_	1.2758**	0.4576	w*L	β_7_	-0.0024	0.0431
C	β_3_	0.9396***	0.2850	w*C	β_8_	-0.0399	0.0265
w^2^	β_4_	-0.0068	0.0100	L*C	β_9_	-0.1881**	0.0715
σ^2^		0.1486***	0.0329	γ		0.9367***	0.0247
μ		-0.7462***	0.2155				
log likelihood function			124.0968	LR test			31.5077

^**a**^ Statistical significance is denoted by

*** for 0.5% level,

** for 1% level, and

* for 5% level.

As is show from Table 5, water, labor and capital input have passed the significant test of 5%, 1% and 0.5% respectively, and the sign is positive. It shows that the three inputs have a significant positive impact on the farmers' output. The gamma value is 0.9367, and through the hypothesis test at 0.5% level. It shows that the technical error mainly stems from technical inefficiency, accounting for 93.67% of the synthetic error. The remaining part is mainly caused by farmers’ non-controllable factors, accounting for 6.37%.

According to formula (8), we can calculate farmers’ TAE. The estimation results of farmers’ production technique efficiency and TAE are shown in Table 6. The table shows the frequency distribution of farmers’ production technique efficiency in comparison with TAE. The results show that farmers’ average production technical efficiency was 87.97%, and mainly distributed in the range of 0.9 to 1, accounting for 60.79% of the total sample. It shows that farmers have similar agricultural production environment, and there is almost no difference in their production technology. Therefore, farmers’ production technique efficiency distribution is relatively concentrated. In contrast, farmers’ TAE are far lower than the production technique efficiency, with an average of 63.98%. It shows that water resources have a savings potential of 36.02% under the condition that the output and other production conditions remain unchanged. In addition, the frequency distribution of farmers’ TAE also shows a trend of fluctuation, which is distributed in each section. It may be caused by farmers’ own factors, family factors and some other factors. Therefore, we empirically analyzed the factors influencing farmers’ TAE.

**Table 6:** Farmers’ production technique efficiency and technology adoption efficiency.

	Production technique efficiency		Technology adoption efficiency (TAE)	
	Observations	Frequency(%)	Observations	Frequency(%)
(0,0.2)	0	0.00	15	5.40
[0.2,0.3)	0	0.00	9	3.24
[0.3,0.4)	0	0.00	6	2.16
[0.4,0.5)	0	0.00	22	7.91
[0.5,0.6)	6	2.16	27	9.71
[0.6,0.7)	15	5.40	66	23.74
[0.7,0.8)	17	6.12	98	35.25
[0.8,0.9)	71	25.54	33	11.87
[0.9,1)	169	60.79	2	0.72
Min	0.5622		0.1141	
Max	0.9842		0.9415	
Average	0.8797		0.6398	

### Interaction between social network and extension service

According to formula (6), we introduce extension service index, social network index and other factors to Model 1, and introduce extension service index, four dimensions indexes of social network and other factors to Model 2. We introduce the cross item of extension service index and social network index to model 3 on the basis of model 1, and introduce the cross items of extension service index and four dimensions indexes of social network to model 4 on the basis of model 2 (Table 7).

**Table 7:** Results of interaction effects.

Variable	Model 1		Model 2		Model 3		Model 4	
	Coef.	Std.Err.	Coef.	Std.Err.	Coef.	Std.Err.	Coef.	Std.Err.
ES	0.1561***	0.0556	0.1664***	0.0556	0.6068***	0.1416	1.2087***	0.3343
SN	0.2130***	0.0571	—		0.8757***	0.2002	—	
learning	—		0.0843	0.0490	—		0.4727**	0.1709
interaction	—		0.1618***	0.0515	—		0.3030	0.1821
reciprocity	—		0.0845	0.0680	—		0.4035	0.2225
trust	—		0.1174*	0.0562	—		0.5205**	0.1996
SN*ES	—		—		-1.0968***	0.3181	—	
learning*ES	—		—		—		-0.6316*	0.2686
interaction*ES	—		—		—		-0.2383	0.2892
reciprocity*ES	—		—		—		-0.5336	0.3692
trust*ES	—		—		—		-0.6925*	0.3169
sex	0.0210	0.0217	0.0305	0.0225	0.0273	0.0213	0.0371	0.0222
age	-0.0010	0.0011	-0.0007	0.0011	-0.0010	0.0011	-0.0008	0.0011
education	0.0684***	0.0094	0.0679***	0.0094	0.0718***	0.0092	0.0709***	0.0093
water users association	0.0333	0.0191	0.0317	0.0190	0.0296	0.0188	0.0309	0.0189
water resource scarcity awareness	0.0232**	0.0085	0.0210*	0.0086	0.0208*	0.0084	0.0190*	0.0084
water-using environment	0.0269**	0.0101	0.0277**	0.0101	0.0321***	0.0100	0.0317***	0.0100
water price	0.0343***	0.0072	0.0337***	0.0072	0.0359***	0.0070	0.0361***	0.0071
technology cognition	0.0234*	0.0104	0.0232*	0.0103	0.0257*	0.0102	0.0251*	0.0101
_cons	-0.0692	0.1003	-0.2115	0.1176	-0.3674***	0.1309	-0.8557***	0.2311
LR chi2	98.69		103.12		110.33		115.88	
Prob > chi2	0.00		0.00		0.00		0.00	
Log likelihood	129.3371		131.5534		135.1586		137.9344	

^**a**^ Statistical significance is denoted by

*** for 0.5% level,

** for 1% level, and

* for 5% level.

Table 7 reports the basic results. The values of Prob > chi2 of the four models are all 0.00, which shows that the models fit well. Then we comparative analyze the LR chi2 value and the Log likelihood value of the four models. We find that they are bigger in model 3 than in model 1. We also find the same conclusion between model 4 and model 2. It means that the models fit better after introducing the cross items into the model. In addition we find that the coefficients, symbols and significance of each control variables are basically consistent in four models, indicating that the models have good robustness.

**In Model 1**, the coefficients of all parameters except age are positive. It shows that these factors have positive effect in improving farmers’ TAE. The coefficients of social network and extension service are both tested by 0.5% significance, and the coefficients of education, water resource scarcity awareness, water-using environment, water price, technology cognition are significant at 0.5%, 1%, 1%, 0.5% and 5% levels respectively.

The results above represent that social network and extension service are beneficial to improve farmers’ TAE. The higher the farmer's social network stock and the more recognized the extension service, the more farmers’ TAE is. It is mainly because social network and extension service are two main accesses to agricultural technology information for farmers. It is a process of learning agricultural technology whether through social network or extension service. It is beneficial for farmers to better obtain the technology, accumulate experiences and make the best inputs combination. Therefore, both are beneficial to improve farmers’ TAE.

The result of **Model 2** shows that the four dimensions of social network are all have positive effects on farmers’ TAE, and the bigger the indexes, the higher farmers’ TAE. From the point of the significance, interaction and trust are examined by significance tests of 0.5% and 5% respectively, while learning and reciprocity is not significant. The possible reason of the above result is that learning has the effect of knowledge spillover [23], which can promote learning. Farmers adjust the agricultural input through learning [7], to improve the efficiency of technology adoption. However, learning failed the significant test, which may be due to the failure to examine the information shared by others. Farmers acquire technical information through interaction to study, which can effectively reduce the risk uncertainty [8,24]. Trust can reduce the transaction costs and promote cooperation [25]. Social network is formed under a strong sense of long-term responsibility and reflects mutual trust, which are the least easy to disentangle [26]. Through trust, farmers can easily get the most mature experiences from other adopters, which are more conducive to improve TAE. The relationship between social network members of modern economic society is the mutually beneficial relations of cooperation, joint and coordinated [27, 28]. The reciprocity relations can help individuals to pursue their own interests while taking into account the interests of others, to achieve the improvement of their own interests and the interests of others [29], which is beneficial to farmers to improve the efficiency of technology adoption. However, due to the limitation of farmers' knowledge level, the effect of the reciprocity relations on agricultural production is not ideal. This is also the possible cause of the no significant effect of reciprocity on farmer’s TAE.

The result of **Model 3** shows that social network and extension service have positive influential and significant in 0.5%. It means that both the two kinds of access to agricultural technology information can improve farmer’s TAE, which further verified the result of Model 1. The coefficient of the cross item of social network and extension service is negative, and it is tested by 0.5% significance. It shows that social network and extension service has the substitution effect in improving farmer’s TAE. With the increase of social network, the positive influential of extension service on farmer’s TAE weakens. Or, along with the extension service enhancement, the improvement of social network on farmers’ TAE is weakening. Which is to say that **social network and extension service are competitive in improving farmer’s TAE**. Compared with extension service, social network is likely to be more acceptable to farmers. Farmers tend to trust people among their social network such as families, friends and relatives, other than extension staffs. That is called acquaintance culture. When the two promoting ways exist at the same time, especially when the promotion content is inconsistent, farmers will definitely choose social network. So the interaction effects between social network and extension service are negative, which is to say that their relationships on improving farmers’ TAE are comparative.

From the perspective of social network structure (**Model 4**), the coefficients of four dimensions of social network and extension service are positive, and the extension service in 0.5% level through the examination, learning and trust are both significant at 1%. After introducing the cross items, the cross items of learning and trust with extension service are both significant at 5% level, while the cross items of interaction and reciprocity with extension service are not significant. By the way, the coefficient of the four cross terms are all negative. That is to say, the effects between these two dimensions of social network and extension service are substitutions in improving the technology efficiency. Let me put it another way, the improvement effects of learning and trust to farmers’ TAE are both weaken with the increase of extension service. **So learning and trust are also competitive with extension service in improving farmer’s TAE.** The possible reason is that farmers have unconditional trust to their social network members (They might be their families, relatives or close friends). They usually accept and simply copy their experiences without judging, which decrease the final effects.

### Other determinants of farmers’ TAE

Besides social network and extension service, we also focus on some other factors’ effect on famers’ TAE such as water users association, water resource scarcity awareness, water-using environment, water price, technology cognition. The effect of these factors on farmers’ TAE has all been verified in the model (Table 7).

The establishment of water users association is conducive to the management and maintenance of water conservancy facilities, water resources allocation and water disputes reducing. On the other hand, the establishment of water users association also is beneficial to improving farmers' water resources utilization awareness, so it can improve farmers’ TAE to establish water users association. Previous studies have showed that water users association positively affects famers’ TAE [14].

The higher the farmers’ water resource scarcity awareness, the more beneficial farmers’ TAE is. With the environment awareness raising, the scarcity of resources is more and more profound understanding by farmers. Especially in Minqin, special continental desert climate features make the generations of people living here to fight with the environment. A strong awareness of the water crisis drive farmers to save water, love water, and carefully use every drop of water to improve the utilization efficiency of water resources.

Improving the water-using environment can also significantly improve farmers’ TAE. The more the phenomenon of stealing water, the less guarantee of water using for farmers, and the worse of farmers’ TAE. In addition, the occurrence of water theft is certainly based on the destruction of irrigation facilities, and the damaged of irrigation facilities will lead to increasing maintenance and management costs, the wasting of water resources, and farmers' affection. It is not good for improving farmers’ TAE. So the less the phenomenon of water theft, the better the water environment, the higher the farmers’ TAE.

Water price is a regulator of farmers’ consumption of water resources. Reasonable water price can optimize the allocation of water resources, and alleviate the contradiction between water shortage and large demand for water resources. Farmers are rational individuals. Water price makes farmers concern about water resources management. When water price is higher than the reasonable price in mind, farmers will decrease water consumption and find ways to improve the utilization efficiency of water resources. So water price has a positive effect on farmers’ TAE.

The deeper the farmers’ technology cognition, the more beneficial the farmers’ TAE is. Improving farmers’ TAE cannot simply rely on saving water. We also must ensure crop production. The traditional theory holds that water saving and crop yield are irreconcilable contradictions. In fact, WSIT can achieve the goal of water saving and high yield at the same time. Then, the more farmers know about WSIT, the more skillful they are, the more conducive to achieving the purpose of water saving and production increasing. Of course, it is beneficial to improve farmers’ TAE.

## Conclusion and discussion

### Conclusion

Based on the survey data of farmers’ adoption of WSIT in Minqin, we calculate farmers’ TAE and analyze the interactive effects of social network and extension service on farmers’ TAE. The conclusions present as following: First, both social network and extension service increase farmer TAE, but it is competitive relationship between them. Second, as for the four dimensions of social network, interaction and trust have positive effects on farmers’ TAE, and the relationship between learning and extension service is competitive, so as trust. Third, beside social network and extension service, there are also many other factors significantly affect farmers’ TAE, such as water users association, water resource scarcity awareness, water-using environment, water price and farmers’ technology cognition.

The policy implications of the above conclusions are far-reaching. First, farmers’ social learning and demonstration effect should be paid much more attention to. Series of measures should be taken to cultivate farmers’ social network during agricultural technology promotion. The functions of social network including information dissemination and access, risk aversion etc., help farmers accumulate knowledge of agricultural technology and improve TAE through learning. Second, extension service should be improved to satisfy farmers’ multiple demand on agricultural technology. As one of the most effective promotion method, extension service can help the government to promote new agricultural technology quickly and help farmers to learn how to use a new agricultural technology correctly. So government should innovate their promotion methods to improve promotion quality, and thinking about the technology needs from the eyes of farmers. Third, government should apply different promotion methods to different regions. Specifically, they should increase the intensity of extension service for the remote villages or the villages with ancient and old-fashioned social network. For example, they can improve TAE through technical guidance and training from extension agency and extension staffs. While for the rural areas closer to town and with developed social network, we should focus on fostering the demonstration households, and promote agricultural technologies and improve the TAE through the demonstration of typical farmers. In addition, government should also pay attention to the using of other ways, such as water price, water rights management and technology publicity, etc., so as to enable the government’s extension service to play a more effective role in improving farmers’ TAE.

### Discussion

As two main accesses to farmers’ agricultural technology information, the significant importance of social network and extension service to farmers’ TAE has been proved [3–5]. While their interactive effects between them have not been paid much attention to. The purpose of our paper is to figure out the interactive relationship of social network and extension service in improving farmers’ TAE. By establishing a two steps measurement to calculate famers’ TAE, four Tobit models were applied to comparatively analyze the interactive effects. We found that social network and extension service are competitive in improving farmers’ TAE both from the perspective of overall social network and the perspective of four dimensions of social network.

Our finding shows that the interactive effects of social network and extension service on farmers' technology adoption efficiency are negative significantly. It is mainly due to the acquaintance culture [30–32]. Farmers tend to trust their social networks, such as their families, relatives and friends, rather than extension staffs. Thus, as two main promotion strategies, social network is more likely to be acceptable to farmers than extension service. However, the simple copying of others’ experiences without judging will inevitably be difficult to fit their own situation. So the interactive effects of social network and extension service are competitive on farmers’ TAE. But different from ours, the finding of Genius et al. suggest that both extension services and social learning are strong determinants of technology adoption, while the effectiveness of each type of informational channel is enhanced by the presence of the other [2]. The different conclusions may be caused by the following reasons. First, social learning and extension service may help each other to make the adoption decision. But it will hardly for them to cooperate with each other to improve adoption efficiency. Adoption decision is a binary choice, while adoption efficiency may be affected by inappropriate experience from others. Second, social networks are rich in content, including learning, interaction, reciprocity and trust. Genius et al. analyze the subject only from social learning. That is another reason why our results are different.

There are still some deficiencies in our research. The improving of farmers’ TAE is a dynamic process. Farmers can raise technology cognition, accumulate experiences and avoid risks through social network gradually. So it will definitely be better if our research can be studied in a long-term range. Nevertheless, our study still has its positive significance. The findings suggest government to apply different promotion methods in different regions due to the negative interactive effect between social network and extension service. It may shed novel light on government’s new agricultural technology promotion strategy to improve farmers’ TAE in developing countries.

## Supporting information

S1 File (DOCX)
