# Supplementary material for: Social network and extension service in farmers’ agricultural technology adoption efficiency
Source: PLoS One. 2020 Jul 10;15(7):e0235927. doi: 10.1371/journal.pone.0235927 (PMC7351193; doi:10.1371/journal.pone.0235927)
Supplement: S1 File — (DOCX) [file pone.0235927.s001.docx]

**农户节水灌溉技术采用调查**

**A Survey on farmers’ water-saving irrigation technology adoption**

调查所收集的信息将用于民勤地区节水灌溉技术采用的实证研究。该研究旨在为技术推广提供新思路，以期提高农户技术采用效率。调查采取不记名形式，数据仅作为科学研究使用。您的信息和隐私是受保护的。感谢您的支持与配合。

The information gathered through this questionnaire will be used as part of empirical research into farmers’ water-saving irrigation technology （WSIT） adoption in Minqin Area. The research is conducted for new promotion strategy to improve farmers’ WSIT adoption efficiency. The survey is anonymous, and the data is used for scientific research only. Your information and privacy are protected. Thank you.

编号：_______________ 调查员：_________________ 调查日期：2018年/____月/ ____日

NO.：________________ Investigator: ________________ Date: ____ /____ /2018 (mm/dd/yyyy)

调查地点：____________省____________市（县）___________镇（乡）_____________村

Survey location (Village/ County/ City/ Province): __________________________________________________

1.基本特征/ General information

1.1个体信息/Individual Information

（1）性别（）

What is your sex? ()

A.男/ Male B.女/ Female

（2）年龄_____岁。

How old are you? _____ years

（3）您是户主吗？（）

Are you a household head? ()

A.是/Yes B.否/No

如果不是户主？户主的年龄是_____岁，性别是（）

If you are not the household head, how old is the household head? _________ years

What gender is the household head?

A.男/ Male B.女/ Female

（4）您的文化程度是？（）

What is your high qualification? ()

A.不识字或识字很少/ Illiterate B.小学/Primary school C.初中/Middle school

D.高中（含中专）/High school E.大专及以上/College and above

（5）从事农业生产_____年。

How many years of farming experience do you have? _____years

1.2家庭特征/ Family characteristics

（1）家庭人口情况/：您家有人口数____人，男性劳动力_____人，女性劳动力有_____人，非劳动力有____人。

Family size: Numbers of people in your family is _____. Numbers of male labor is _____. Numbers of female labor is _____. Numbers of non-labor is _____.

（2）家庭耕地情况/Cultivated information

| 家庭耕地面积（亩）  Arable land area (mu) |  | 租入亩数（亩）  Area of rented land (mu) |  | 灌溉面积（亩）  Irrigation area (mu) |  |
| --- | --- | --- | --- | --- | --- |
| 家庭耕地块数  Number of plots |  | 最大一块地面积（亩）  Area of the largest one (mu) |  | 最小一块地面积（亩）  Area of the smallest one (mu) |  |

（3）2017年您家总收入_____元。其中，种植收入_____元，水利补贴_____元。

Annual household income of your family in 2017 is _____ yuan. Among them, planting income is _____ yuan, and irrigation subsidy is _____ yuan.

（4）2017年您家总支出_____元。其中，种植支出_____元；灌溉支出_____元；人情礼品支出_____元；教育支出_____元。

Annual household expend of your family in 2017 is _____yuan. Among them, planting expend is _____ yuan, irrigation expend is ____ yuan, the spending on interpersonal interaction is ____ yuan, and education expend is ____ yuan.

（5）您家是否参加了农业合作社?（）

Did your family participate in an agricultural cooperative? ()

A.是/Yes B.否/No

（6）您家是否加入了用水者协会?（）

Did your family participate in a water users association? ()

A.是/Yes B.否/No

如果没有加入，原因是（）？

If your answer is No, then why didn’t you participate in? ()

A.本村没有/There is no one in my village.

B.本村没有但我不愿参加/ I do not want to though there is one in the village.

（7）您家获得农业贷款容易吗（）

Is it easy for your family to get an agricultural loan? ()

A.很难贷/Very difficult B.不容易/Not that much easy C.一般/Easy

D.容易/ Quite easy E.很容易/ Extremely easy

1.3村庄特征/ Village characteristics

（1）你们村大概有_______户农户。

How many households are there in your village? __________

（2）您所在村的主要地形是什么？（）

What is the main terrain of your village? ()

A.平原/Plain B.山地/Mountain C.丘陵/Hill

（3）你所在村离红崖山水库的距离是__________公里

How far is your village from Hongyashan Reservoir? __________ km

（4）你所在村离最近的市场的距离是__________公里

How far is your village from the nearest market? __________ km

（5）你所在村离最近的车站的距离是__________公里

How far is your village from the nearest bus stop? __________ km

（6）你所在村离镇上的距离是__________公里

How far is your village from the town? __________ km

（7）你们村里有用水协会吗？（）

Is there any water users association in your village? ()

A.是/Yes B.否/No

（8）你们村里有农技推广人员吗？（）

Is there any extension staff in your village? ()

A.是/Yes B.否/No

2.农业生产和灌溉情况Planting and irrigation information

（1）种植作物投入产出情况（2017年）/The input and output of planting in 2017

| 作物名称  Crop name |  |  |  |  |  |
| --- | --- | --- | --- | --- | --- |
| 种植面积（亩）  Planting area (mu) |  |  |  |  |  |
| 灌溉面积（亩）  Irrigation area (mu) |  |  |  |  |  |
| 节水灌溉面积  Water-saving irrigation area (mu) |  |  |  |  |  |
| 单产（公斤）  Yield (kg) |  |  |  |  |  |
| 出售（公斤）  Sold (kg) |  |  |  |  |  |
| 售出单价（元/公斤）  Selling price (yuan / kg) |  |  |  |  |  |
| 种苗投入（元/亩）  Applied seeds (yuan / mu) |  |  |  |  |  |
| 农药（元/亩）  Applied pesticide (yuan / mu) |  |  |  |  |  |
| 化肥（元/亩）  Applied fertilizers (yuan / mu) |  |  |  |  |  |
| 农家肥（元/亩）  Applied compost (yuan / mu) |  |  |  |  |  |
| 雇工（元/亩）  Hired labors (yuan / mu) |  |  |  |  |  |
| 灌溉水（元/亩）  Applied irrigation water (yuan / mu) |  |  |  |  |  |
| 电费（元/亩）  Applied electricity (yuan / mu) |  |  |  |  |  |
| 机械租赁（元/亩）  Applied machines (yuan / mu) |  |  |  |  |  |
| 其他（元/亩）  Others (yuan / mu) |  |  |  |  |  |

（2）您家的灌溉用水是否完全来自地下水？（）

Is the irrigation water of your family all from groundwater? ()

A.是/Yes B.否/No

（3）您所在村的机井是不是越打越深了？（）

Are wells in your village getting deeper and deeper? ()

A.非常浅/ Strongly disagree B.很浅/ Disagree C.一般/ The same as usual

D.很深/ Agree E.非常深/ Strongly Agree

（4）您所在村子有是否有偷水现象？（）

Does water theft occur in your family? ()

A.是/Yes B.否/No

（5）您所在村子用水纠纷多吗？（）

Is the phenomenon of water dispute becoming less and less common in your village? ()

A.特别多/ Strongly disagree B.比较多/ Disagree C.一般/ The same as usual

D.偶尔有/ Agree E.从来没有/ Strongly Agree

（6）你认为水价贵吗？（）

Is water price become more and more expensive? ()

A.一点都不贵/Strongly disagree B.不贵/Disagree C.没感觉/The same as usual

D.很贵/ Agree E.非常贵/ Strongly agree

（7）您所在村庄农业用水管理政策是（）

What is the irrigation water management policy in your village? ()

A.定额管理，超额高水价/ Quota management and excess usage will charge a high price

B.没有定额，水价都一样/ Charge according to usage and the price remains the same

C.实行定额管理，给予节水者奖励/ Quota management and give rewards to water-savers

（8）您认为最合适的水费收取标准是哪种？（）

Which do you think is the best way to charge for irrigation water? ()

A.用水量/ Water consumption B.灌溉面积/ Irrigation area

C.灌溉时间/Irrigation time D.其他/Other, ____________

（9）如果水价上涨，您是否会因为水价过高而酌情减少灌溉次数？（）

Will you choose to decrease the times of irrigation if water price rising? ()

A.是/Yes B.否/No

（10）如果水价上涨，您是否会因为水价过高而选择节水技术？（）

Will you choose to adopt WSIT if water price rising? ()

A.是/Yes B.否/No

（11）如果水价上涨，您是否会因为水价过高而选择增加节水作物种植？（）

Will you choose to plant more drought-tolerant crops if water price rising?

A.是/Yes B.否/No

（12）您家是否在使用节水灌溉技术？（）

Does your family adopt WSIT? ()

A.是/Yes B.否/No

若是，使用的是哪种技术（）？未来最期望使用什么技术？（）（可多选）

If the answer is yes, what they are? () Which technology do you want to continue adopting in the future? () (Multiple choice)

A.滴灌/Drip irrigation B.渗灌/ Seepage irrigation

C.喷灌/ Sprinkler irrigation D.微灌/ Micro irrigation

E.低压管灌/Low pressure pipe irrigation F.其他/ Other _______

3.节水灌溉技术采用情况/ Information about WSIT adoption

3.1节水灌溉技术认知/Cognition

（1）您获取的农业技术信息来源是什么？（）

How can you obtain agricultural technology information? ()

A.县乡农技人员田间指导/Field training from extension staff

B.跟周围农民看样学习/Learning from the farmers around

C.电视、书刊、报纸/ TVs, magazines, newspapers, et al.

D.科技博览会或技术推介会/Technology expo or technology promotion conference

E.网络/Internet

F.手机/Cellphone

G.自己摸索/Learning by doing

H.其它Others such as _____

（2）您是否听说过以下节水灌溉技术（如滴灌、渗灌、喷灌、微灌、低压管灌）（）？

Do you ever hear about the following WSIT, such as drip irrigation, seepage irrigation, sprinkler irrigation, micro irrigation, low pressure pipe irrigation? ()

A.是/Yes B.否/No

如果是，您是通过何种方式知道的？（）

If the answer is yes, how do you know? ()

A.电视广播、书报、网络等媒体/Medias such as TV, newspaper, Internet, et al.

B.农技部门、科研单位、合作社、企业/Extension agency, research department, cooperatives, enterprises.

C.商家推荐/Recommendation from salesman

D.熟人推荐、其他人选择/ Recommendation from acquaintance or following the choice of others

E.其他/ other way ____________________________

（3）您认为节水灌溉技术对保障粮食生产重不重要？（）

Is it important for ensuring agricultural production to adopt WSIT? ()

A.非常不重要/ Extremely unimportant B.不重要/ Quite unimportant

C.一般/Neither important or unimportant D.比较重要/Quite important

E.非常重要/ Extremely important

（4）您对节水灌溉技术的了解程？（）

To what extent do you know about WSIT? ()

A.很不了解/ Not at all B.不了解/ A little bit C.一般 somewhat

D.比较了解/Quite a bit E.非常了解/ A tremendous amount

（5）您对节水灌溉政策的了解程度? （）

To what extent do you know about the policy of water-saving irrigation? ()

A.很不了解/ Not at all B.不了解/ A little bit C.一般 somewhat

D.比较了解/Quite a bit E.非常了解/ A tremendous amount

（6）您认为节水灌溉技术的主要功能是什么？（）（可多选）

What do you think the function of WSIT is? () (Multiple choice)

A.节水/Saving water B.增产/ Increasing production C.增收/ Increasing Income

D.提高生产效率/Improving Production efficiency E.其他/ Other function, __________

（7）您认为较传统灌溉方式，节水灌溉技术的效果如何？（）

How do you think about the effect of WSIT compared with traditional irrigation? ()

A.比传统技术差很多/Much worse B.比传统技术差点/Worse

C.没差别/About the same D.比传统技术好点/Better

E.比传统技术好很多/Much better

3.2节水灌溉技术采用意愿与支付意愿/Willingness to adopt and willingness to pay

（1）您对一项新型农业技术的采用态度是什么？（）

What will you do if a new agricultural technology appears? ()

A.有新技术，马上采用/I will adopt immediately if a new agricultural technology appears

B.看看效果，稍后采用/I will adopt after taking a look at its effect

C.其他人都采用了，我再采用/I will adopt if others did

（2）在决定是否采用一项农业新技术时，您认为以下因素中哪一项是最重要的？（）

Which of the following factors do you think is the most important when you decided to adopt a new agricultural technology? ()

A.技术接受难易程度或能不能得到技术支持/Is the technology easy to master, or whether there is technique support.

B.采用技术的资金来源或能不能得到资金支持/Source of the funding for adopting technology, or whether there is financial support.

C.技术使用所带来的经济效益或生产出来的产品好不好卖/Economic benefits brought by technology adopting, or whether the productions are sold well.

D.其他人有没有采用/Whether others adopted。

E.其它/Others ______________________

（3）您是否愿意采用节水灌溉技术？（）

Do you want to adopt WSIT? ()

A.非常不愿意/Quite unwilling B.不愿意/Unwilling C.一般/ Neutral

D愿意/Willing E非常愿意/Quite willing

若不愿意，原因有哪些？（）

If you do not want to, what are the reasons? ()

A.麻烦/ It's troublesome

B. 土地面积小地块分散，不好用/Small plots of land scattered and difficult to use

C.效果差/Poor effects

D. 投资大预期回报低/Big investment, low expected return

E. 设备易坏，维修困难/Equipment is unstable and difficult to maintain

F.其他/Other reason ______

（4）如果让您采用节水灌溉技术，您最多愿意为此支付_________元。

How much are you maximum willing to pay if you are asked to adopt WSIT? _______ yuan.

3.3节水灌溉技术采用/WSIT adoption

（1）您所在村庄是否有推广节水灌溉技术？（）

Does WSIT promoted in your village? ()

A.是/Yes B.否/No

（2）村里是否有示范户或用水者协会指导学习节水灌溉技术？（）

Are there model households or associations who teach how to use WSIT in the village? ()

A.是/Yes B.否/No

（3）你家是否采用节水灌溉技术？（）

Does your family adopt WSIT? ()

A.是/Yes B.否/No

**如果未采用，请回答问题（4）～（5）；否则，请回答问题（6）～（11）。**

**If your answer is No, please complete question (4)-(5). Otherwise, go to question (6)-(11).**

（4）您家没有采用节水灌溉技术的原因是什么？（）

What are the reasons for not adopting? ()

A.技术太复杂，学不会/Too complicated to master

B.前期投资太大/Big upfront investment

C.地块不适用/Small plots of land scattered and difficult to use

D.增产增收不明显/ No significant effect on increasing production and income

E.灌溉效果差/ Poor irrigation effect

F.后期维护成本高/ High post-maintenance costs

G.其他/ Other reasons _____________________

（5）您家未来是否打算采用节水灌溉技术？（）

Do you plan to adopt WSIT in the near future? ()

A.是/Yes B.否/No

（6）您家采用节水灌溉的原因是什么？（）

What are the reasons for adopting? ()

A.农技推广人员推广/ Promotion from extension staff

B.政府示范村Learning from model village

C.自发采用/ Spontaneous adoption

D.农业合作社建议/ Advices from cooperatives

E.高校、科研机构推广/ Recommendation from college or research department

F.别人采用了，我也跟着采用/ I adopt because others adopted

G.企业推荐/ Recommendation from enterprises

H.其他/ Others reasons _______

（7）您家的节水灌溉设施总投资______元。其中，家庭出资______元；其他费用由（）出资（可多选）。

How much is the total investment of your water-saving irrigation equipment? ______yuan

Among them, how much does your family spend? ______yuan

Who provide the other money? () (Multiple choice)

A.政府/Government B.村委会/ Village committee

C.农业合作社/Cooperatives D.其他/ Others such as ____

（8）政府对采用节水灌溉技术有补贴吗？（）

Does the government subsidize for adopting WSIT? ()

A.是/Yes B.否/No

若有，补贴方式是什么？（）

If yes, how does the government subsidize? ()

A.资金/Money B.设备/Equipment C. 技术支持/Technical Support

（9）您家节水灌溉设施的提供方式是哪种？（）

Which of the following is the providing method of your family’s WSIT equipment? ()

A.政府出资建设经营/ Government-funded construction and operation

B.私人投资建设经营/ Individual-funded construction and operation

C.政府建设私人承包经营/Government-funded construction and individual-contracting operation

D.村民合作建设共同经营/Villagers' cooperative construction and joint operation

E.政府私人共同建设经营Government and individual funded construction and operation

（10）您家节水灌溉设施的维护单位是?（）

Who is going to maintain WSIT equipment of your family if it is broken? ()

A.灌区管理局/ Administration of the Irrigation District B.农户个人/ Farmers themselves

C.村委会/ Village committee D.用水者协会/Water users association

E.不维护/Not maintained F.其他/Others such as_____

（11）您家节水灌溉设施维修是否及时？（）

Is the water-saving irrigation equipment of your family maintained in a timely manner? ()

A.很不及时/Extremely untimely B.不及时/ Quite untimely C.一般/Moderately timely

D.很及时/ Quite timely E.很及时/ Extremely timely

3.4节水灌溉技术采用评价/Adoption evaluation

（1）您对采用节水灌溉技术后的效果满意吗？/（）

To what extent you are satisfied with the effect of adopting WSIT?

A.很不满意/Quite dissatisfied B.比较不满意/ Dissatisfied

C.一般/Neither dissatisfied or satisfied D.比较满意/ Satisfied

E.非常满意/Quite satisfied

不满意的原因是______________________________________________

The reasons why dissatisfied are __________________________________

（2）您在多大程度上同意下列有关使用节水灌溉技术后的说法，1=非常不同意，2=不同意，3=一般，4=同意，5=非常同意。

To what extent do you agree with the following statements after adopting WSIT? 1= Extremely disagree, 2= Quite disagree, 3= Neither disagree or agree, 4= Quite agree, 5= Extremely agree

| 测量题项  Statements | 1 | 2 | 3 | 4 | 5 |
| --- | --- | --- | --- | --- | --- |
| 较传统技术作物产量提高了  The production increased after adoption |  |  |  |  |  |
| 较传统技术种植收入提高了  The income increased after adoption |  |  |  |  |  |
| 节水灌溉技术能够节约水、土资源  Water and land resources can ben saved after adoption |  |  |  |  |  |
| 节水灌溉技术所需劳动力减少  Labors can ben saved after adoption |  |  |  |  |  |
| 节水灌溉技术采用后水费减少了  Irrigation water bill decreased after adoption |  |  |  |  |  |
| 灌溉用水紧缺情况改善了  The situation of irrigation water shortage improved after adoption |  |  |  |  |  |
| 用水纠纷明显减少  Water disputes reduced significantly after adoption |  |  |  |  |  |
| 政府提供的扶持政策（如补贴、设施等）是有用的  Government’s promotion policy such as subsidy, equipment supplying, et al. is useful |  |  |  |  |  |
| 农技部门提供的技术培训、技术信息是充分的  The technical training and technology information provided by extension agency are sufficient |  |  |  |  |  |
| 与农技部门技术交流是便利的  It is convenient to contact with extension agency |  |  |  |  |  |
| 我的邻居及亲朋好友中有很多采用节水灌溉技术的  Many of my friends and relatives adopted WSIT |  |  |  |  |  |
| 节水灌溉技术采用经历比期望的更好  The experience of WSIT adoption is better than expected |  |  |  |  |  |
| 节水灌溉技术在当地能持续使用  WSIT can be continuously adopted locally |  |  |  |  |  |
| 我将继续采用节水灌溉技术  I will continuously adopt WSIT |  |  |  |  |  |
| 我将推荐朋友采用节水灌溉技术  I will recommend my friends and relatives to adopt WSIT |  |  |  |  |  |

4.技术推广/Extension service

4.1推广方式/Ways of extension service

（1）您家接受过哪种形式的节水灌溉推广服务？（）（可多选）

Which of the following ways of extension service your family has taken? () (Multiple choice)

A.专家现场技术指导/ Field technical guidance

B.专家集中培训/Collective technical training

C.资料宣传/Information promotion

D.咨询服务/ Consultation service

E.电视讲座/ TV Lecture

F.广播报刊宣传/ Broadcasting or newspaper promotion

G.网络资料/ Internet information

H.其他/ Other ways such as __________________________

（2）您参加节水灌溉推广服务的频率如何？1=从不；2=很少；3=一般；4=经常；5=总是

How often does your family participate in the extension service? 1= Almost never, 2= Once in a while, 3= Sometimes, 4= Frequently, 5= Almost all the time.

| 测量题项  Statements | 1 | 2 | 3 | 4 | 5 |
| --- | --- | --- | --- | --- | --- |
| 专家现场技术指导  Field technical guidance |  |  |  |  |  |
| 专家集中培训  Collective technical training |  |  |  |  |  |
| 资料宣传  Information promotion |  |  |  |  |  |
| 咨询服务  Consultation service |  |  |  |  |  |
| 电视讲座  TV Lecture |  |  |  |  |  |
| 广播报刊宣传  Broadcasting or newspaper promotion |  |  |  |  |  |
| 网络资料  Internet information |  |  |  |  |  |

4.2推广效果/Effects of extension service

（1）您家对推广服务满意吗？（）

How satisfied are you with the government’s extension services? ()

A.非常满意/ Extremely satisfied B.满意/ Very satisfied C.一般/ Somewhat satisfied

D.不满意/ Not so satisfied E.非常不满意/ Not at all satisfied

（2）如有节水灌溉技术问题，您是否主动联系农技人员？（）

Do you contact with extension staff actively when you have technique problems on WSIT? ()

A.是/Yes B.否/No

若是，是否能很容易地联系到农技人员？（）

If the answer is yes, can you easily reach the extension staff? ()

A.是/Yes B.否/No

（3）农技人员推广的内容是不是很容易理解？（）

Is it easy for me to understand the content of the extension service? ()

A非常不容易/ Not at all easy B不容易/Slightly easy C.一般/ Moderately easy

D.容易/Quite easy E非常容易/ Extremely easy

（4）农技人员推广的技术是不是很容易掌握？（）

Is it easy for me to master the skill of WSIT through extension service? ()

A非常不容易/ Not at all easy B不容易/Slightly easy C.一般/ Moderately easy

D.容易/Quite easy E非常容易/ Extremely easy

（5）农技人员推广的内容对您的生产生活是否有帮助？（）

Is Government’s extension service of great help to agricultural production? ()

A没有帮助/Not any help at all B帮助不大/Slightly helpful

C.还行/A moderate amount of help D.有帮助/ A lot of help

E.很有帮助/A great deal of help

5.社会网络/ Social network

5.1网络学习/Learning

以下网络学习行为发生的频率如何？1=从不；2=很少；3=一般；4=经常；5=总是

How often does the following learning behavior happen? 1= Almost never, 2= Once in a while, 3= Sometimes, 4= Frequently, 5= Almost all the time.

| 测量题项  Statements | 1 | 2 | 3 | 4 | 5 |
| --- | --- | --- | --- | --- | --- |
| 与他人交流节水灌溉技术使用心得的频率  Communicating with others about technology using |  |  |  |  |  |
| 向技术示范户请教节水灌溉问题的频率  Consulting model household about technology matters |  |  |  |  |  |
| 去技术示范户的田里参观的频率  Visiting demonstration farmland |  |  |  |  |  |

5.2网络互动/Interaction

以下网络互动行为发生的频率如何？1=从不；2=很少；3=一般；4=经常；5=总是

How often does the following interaction behavior happen? 1= Almost never, 2= Once in a while, 3= Sometimes, 4= Frequently, 5= Almost all the time.

| 测量题项  Statements | 1 | 2 | 3 | 4 | 5 |
| --- | --- | --- | --- | --- | --- |
| 与普通技术采用者互动的频率  Interaction with regular technology adopters |  |  |  |  |  |
| 与是亲密好友的技术采用者互动的频率  Interaction with adopters who are intimate friends with each other |  |  |  |  |  |

5.3网络互惠/Reciprocity

您在多大程度上同意下列说法，1=非常不同意，2=不同意，3=一般，4=同意，5=非常同意。

To what extent do you agree with the following statements? 1= Extremely disagree, 2= Quite disagree, 3= Neither disagree nor agree, 4= Quite agree, 5= Extremely agree

| 测量题项  Statements | 1 | 2 | 3 | 4 | 5 |
| --- | --- | --- | --- | --- | --- |
| 家里有事时大家愿意来帮忙  Everyone is willing to help when some general events happen |  |  |  |  |  |
| 遇到困难时有很多人帮忙想办法解决  There are a lot of people who help out during difficult time |  |  |  |  |  |

5.4网络信任/Trust

您在多大程度上同意下列说法，1=非常不同意，2=不同意，3=一般，4=同意，5=非常同意。

To what extent do you agree with the following statements? 1= Extremely disagree, 2= Quite disagree, 3= Neither disagree nor agree, 4= Quite agree, 5= Extremely agree

| 测量题项  Statements | 1 | 2 | 3 | 4 | 5 |
| --- | --- | --- | --- | --- | --- |
| 我愿意借东西给周围人  I'm willing to lend something to people around me |  |  |  |  |  |
| 我们村邻里关系和谐  Neighborhood relations are very harmonious in my village |  |  |  |  |  |

**调查结束，谢谢合作！**

**Thank you for your cooperation!**
